# Supplementary material for: MetaRibo-Seq measures translation in microbiomes
Source: Nat Commun. 2020 Jun 29;11:3268. doi: 10.1038/s41467-020-17081-z (PMC7324362; doi:10.1038/s41467-020-17081-z)
Supplement: Supplementary file 10 — Supplementary Data 7 [file 41467_2020_17081_MOESM10_ESM.zip › File2/Confidence_High_Taxonomy/94640_out.krona.html]

Javascript must be enabled to view this page.

members
magnitude
magnitudeUnassigned
count
unassigned
taxon
rank

94640\_out

4

2
superkingdom
4

phylum
1239
3

3
class
186801


SRS053356\_contig\_number\_45537
order
186802
3
1

2
family
541000

946234
genus
2


SRS051031\_contig\_number\_34835SRS1041145\_contig\_number\_39348
species
1193534
2

phylum
201174
1

class
1760
1

85006
order
1

family
1042322
1

1
genus
1217415

species

SRS077392\_contig\_number\_19088
1638985
1
